# Early Neurotoxic Effects of Inorganic Arsenic Modulate Cortical GSH Levels Associated With the Activation of the Nrf2 and NFκB Pathways, Expression of Amino Acid Transporters and NMDA Receptors and the Production of Hydrogen Sulfide

**DOI:** 10.3389/fncel.2020.00017

**Published:** 2020-02-25

**Authors:** Daniela Silva-Adaya, Lucio Antonio Ramos-Chávez, Pavel Petrosyan, Wendy Leslie González-Alfonso, Alegna Pérez-Acosta, Maria E. Gonsebatt

**Affiliations:** ^1^Departamento de Medicina Genómica, Instituto de Investigaciones Biomédicas, Universidad Nacional Autónoma de México, México, Mexico; ^2^Laboratorio Experimental de Enfermedades Neurodegenerativas, Instituto Nacional de Neurología y Neurocirugía, México, Mexico; ^3^Departamento de Neuroquímica, Subdirección de Investigaciones Clínicas, Instituto Nacional de Psiquiatría Ramón de la Fuente, Ciudad de México, México, Mexico

**Keywords:** arsenic, GSH, Nrf2, NFκB, CNS cysteine/glutamate transporters, H_2_S

## Abstract

Exposure to toxic metals and metalloids is an important cause of preventable diseases worldwide. Inorganic arsenic (iAs) affects several organs and tissues, causing neurobehavioral alterations in the central nervous system (CNS) that might lead to neurodegeneration. In this work, we wanted to explore the time- and dose-related changes on glutathione (GSH) levels in several regions of the CNS, such as the cortex, striatum, hippocampus, and cerebellum, to identify the initial cellular changes associated to GSH depletion due to iAs exposure. Mice received a single intraperitoneal injection containing 5 or 14 mg/kg sodium arsenite. Animals were killed at 2, 6, and 24 h. Significant depletion of GSH levels was observed in the cortex at 2 and 6 h, while on the striatum, hippocampus, or cerebellum regions, no significant changes were observed. GSH depletion in the cortex was associated with the activation of the nuclear factor erythroid 2-related factor 2 (Nrf2) and nuclear factor kappa B (NFκB) pathways, which led to the upregulation of xCT, excitatory amino acid carrier 1 (EAAC1), glutamate/aspartate transporter (GLAST), and glial glutamate transporter 1 (GLT-1), and the activation of the transsulfuration pathways, which led to the overproduction of H_2_S in the cortex and increased levels of GSH in the cortex and cerebellum at 24 h. In the cortex, the *N*-methyl-D-aspartate (NMDA) receptor subunits NR2A and NR2B were also altered at 24 h. These early effects were not homogeneous among different brain regions and indicate early neurotoxic alterations in the cortex and cerebellum.

## Introduction

Improvements in the quality of life of humans is reflected in the increase in our life span; however, environmental pollution continues to be the largest cause of disease and premature death worldwide (Landrigan et al., 2018). The overexploitation of water resources exposes a continuously increasing number of people to toxic elements such as pesticides, nitrites, and metals (Kurwadkar, 2017). Arsenic is an element widely distributed on earth in soil and minerals that, naturally or due to anthropogenic activities, might enter the air, water, or food. Today, human exposure to this toxic element occurs mainly through drinking water or food and represents a worldwide problem affecting over 200 million people. Cardiovascular, endocrine, immune, and neurotoxic effects and several types of cancer have been associated with inorganic arsenic (iAs) exposure (Hong et al., 2014).

Acute neurotoxic effects after oral doses include mild to severe encephalopathy, depending on the dose. Symptoms include confusion, hallucinations, reduced memory, and emotional lability (exaggerated changes in mood or affect; Garza-Lombó et al., 2018a,b, 2019). On the other hand, chronic exposure to iAs is associated with the development of peripheral neuropathies (Garza-Lombó et al., 2019). Furthermore, in children exposed to iAs concentrations ranging from 5 to 50 ppb in drinking water, neurobehavioral alterations such as impaired cognitive functions, verbal abilities, and long-term memory, and decreased motor skills have been reported (Calderón et al., 2001; Parvez et al., 2011). However, it is not clear when the initial effects are observed in the central nervous system (CNS; Garza-Lombó et al., 2019).

iAs crosses the blood-brain barrier and accumulates in different brain regions where it is methylated by As^3+^ methyltransferase, a process that requires thioredoxin or glutathione (GSH) as a reductive agent and *S*-adenosyl methionine (SAM) as the methyl donor (Thomas et al., 2004; Sánchez-Peña et al., 2010). The SAM pathway and GSH production are linked through the transsulfuration pathway (Rodríguez et al., 2005). In addition, regions with higher energy demand, such as the cortex and CA3/CA4 regions of the hippocampus or cerebellum, show the highest GSH levels and thioredoxin immunoreactivity (Sánchez-Peña et al., 2010).

Owing to its high oxygen consumption and low levels of antioxidant enzymes, the brain is vulnerable to the harmful effects of reactive oxygen species (ROS). GSH is the most important endogenous antioxidant in the CNS and plays an important role in the maintenance of the intracellular redox balance and the detoxification of xenobiotics (Gu et al., 2015), being also considered a cysteine and glutamate reservoir in the brain. Intracellular GSH synthesis requires the availability of its precursor amino acids: L-cysteine (L-cys), L-glutamate (L-glu), and L-glycine (L-gly; Robert et al., 2014). Cysteine is the limiting amino acid for GSH; thus, it is continuously imported or synthesized, depending on the cell type. For example, cystine (the oxidized form of cysteine) is taken up into glial cells by the antiporter system xc− (Xc−), which is a Na^+^-independent, Cl-dependent cystine/glutamate exchanger composed of the catalytic subunit xCT and the structural heavy chain 4F2 cell surface antigen (4F2hc). In the brain, the Xc−system is predominately expressed in astrocytes (Bannai, 1986; Robert et al., 2014). On the other hand, a member of the family of excitatory amino acid transporters (EAAT), mainly EAAT3 [excitatory amino acid carrier 1 (EAAC1) in mouse], is an important importer of L-cys in neurons that is present at much lower levels than glutamate or glycine (Aoyama et al., 2012). Therefore, EAAT3/EAAC1 supplies the rate-limiting substrate for GSH synthesis in neurons, while other members of this family, such as EAAT1 or glutamate/aspartate transporter (GLAST) and EAAT2 or glial glutamate transporter 1 (GLT-1), are expressed in glia and participate in the uptake of L-glu (Valdovinos-Flores and Gonsebatt, 2012). Aoyama et al. (2006) reported that EAAC1-knockout mice have decreased neuronal GSH and increased neuronal oxidative stress. These effects were reversed when the animals received the membrane-permeable L-cys precursor *N*-acetylcysteine (Aoyama et al., 2006). We have observed that gestational exposure to iAs through drinking water induced the upregulation of xCT, which was associated with increased levels of GSH in the cortex and hippocampus (Ramos-Chávez et al., 2015; Nelson-Mora et al., 2018). In addition, the overexpression of xCT leads to increased extracellular glutamate and to the downregulation of both *N*-methyl-D-aspartate (NMDA) and α-amino-3-hydroxy-5-methyl-4-isoxazolepropionic acid (AMPA) ionotropic glutamate receptors, with significant negative effects on learning and memory (Ramos-Chávez et al., 2015; Nelson-Mora et al., 2018).

The L-cys required for GSH synthesis in astrocytes can also be generated from methionine through the transsulfuration pathway by the action of cystathionine-β-synthase (CBS) and cystathionine-γ-lyase (CSE; McBean, 2017). In this respect, Coppin et al. (2008) reported CBS upregulation in transformed cultured cells after protracted exposure to iAs that led to increased GSH levels. Moreover, CBS, CSE, and 3-mercaptopyruvate sulfurtransferase (Chen et al., 2004; Singh et al., 2009) are producers of endogenous hydrogen sulfur (H_2_S), a neuromodulator present in the brain that, in excess, could have detrimental effects. CBS is predominantly expressed in the brain and has been associated with the modulation of NMDA receptor (NMDAR)-mediated responses (Huang and Moore, 2015).

Here, we wanted to investigate the early time- and dose-related changes on GSH levels in several regions of the CNS such as the cortex, striatum, hippocampus, and cerebellum, identifying initial cellular changes associated to GSH depletion due to iAs exposure.

## Materials and Methods

### Chemicals and Antibodies

All chemicals were purchased from Sigma–Aldrich (St. Louis, MO, USA) unless otherwise indicated. For Western blots, primary rabbit antibodies against xCT, EAAC1, GLAST, GLT-1, and CBS (Abcam Cat# ab37185, RRID:AB-778944; Abcam Cat# ab124802, RRID:AB-10974334; Abcam Cat# ab416, RRID:AB-304334; Abcam Cat# ab41621, RRID:AB-941782; Abcam Cat# ab135626, RRID:AB-2814659, respectively) were obtained from Abcam (Cambridge, MA, USA). Primary anti-NR2A and anti-NR2B (Millipore Cat# AB1555P, RRID:AB-90770; Millipore Cat# AB1557P, RRID:AB-90772, respectively) antibodies were purchased from Millipore, Bedford, MA, USA. Primary anti-L-type amino acid transporter 1 (anti-LAT1; Santa Cruz Biotechnology Cat# sc-34554, RRID:AB-2270583) antibodies were obtained from Santa Cruz Biotechnology (Santa Cruz, CA, USA). Rabbit antimouse β-tubulin (Sigma-Aldrich Cat# T4026, RRID:AB-477577) was purchased from Sigma-Aldrich.

### Animals and Treatment

Five- to six-week-old male CD-1 mice were obtained from the Animal Care Facility at the Instituto de Investigaciones Biomédicas, UNAM and were maintained at 23–25°C under a 12-h light/dark cycle and relative humidity of 50–60%. Animals had free access to standard food (Harlan 2018S Diet; Harlan, Indianapolis, IN, USA) and water.

Animals were divided into different working groups. To study the acute response, they received an intraperitoneal (i.p.) injection (100 μl) containing 0, 5, or 14 mg of sodium arsenite per kilogram of body weight. Controls received an i.p. injection of 100 μl of isotonic saline solution. Animals were killed at 2, 6, and 24 h after i.p. injection. A semichronic treatment group to compare the acute response was designed. In this group, the animals received i.p. injections containing 0, 2.5, and 5 mg of sodium arsenite per kilogram of body weight per day for 9 days. Sodium arsenite solutions were prepared daily and dissolved in injectable water.

Animals were killed by cervical dislocation, followed by decapitation. Different brain regions were dissected for immunoblotting determination. To determine GSH and/or to generate the membrane-enriched integral protein fractions and to measure H_2_S, tissue samples were immediately homogenized in their respective buffers and kept frozen at −80°C until analysis.

The experiments were performed following the guidelines stated in the “Principles of Laboratory Animal Care” (NIH publication #85-23, revised 1985) and “Especificaciones técnicas para la producción, cuidado y uso de los animales de laboratorio (Clave NOM-062-ZOO-1999)” of the “Norma Oficial Mexicana de la Secretaría de Agricultura, Ganadería, Desarrollo Rural, Pesca y Alimentación (SAGARPA)” (published in August 2001).

### GSH Level Determination

The levels of reduced GSH were measured in the cortex, hippocampus, striatum, and cerebellum using a microplate-adapted fluorometric o-phthalaldehyde (OPA) method (Ramos-Chávez et al., 2015). The method is based on the GSH reaction with o-phthaldialdehyde (OPA) to form a highly stable and fluorescent isoindole derivative. Briefly, wet tissue was homogenized in 10 volumes of ice-cold buffer (154 mM KCl, 5 mM diethylenetriaminepentaacetic acid, and 0.1 M potassium phosphate buffer, pH 6.8). Immediately thereafter, equal volumes of cold acid buffer [40 mM HCl, 10 mM DTPA, 20 mM ascorbic acid, and 10% trichloroacetic acid (TCA)] were added to one volume of homogenate. Two microliters of supernatant was used for GSH determination. Fluorescence was determined with 365 nm excitation and 430 nm emission filters in a DTX 800/880 Multimode Detector (Beckman Coulter, Fullerton, CA, USA).

### Western Blotting

Western blot assays for CBS determination were performed as follows. Total tissue was homogenized in ice-cold lysis buffer [50 mM Tris–HCl, 150 mM NaCl, 2 mM EDTA, 1 mM ethylene glycol tetraacetic acid (EGTA), 2.5 mM sodium pyrophosphate, 1 mM glycerol-2-phosphate, 1 mM sodium orthovanadate, 1% Triton X-100, 1 mM dithiothreitol (DTT), 1 mM phenylmethylsulfonyl fluoride (PMSF), and inhibitor protease cocktail], containing protease inhibitor cocktail. The homogenates were centrifuged at 15, 000× *g* for 15 min at 4°C. Membrane-enriched integral protein fractions were obtained from frozen tissue samples as described by Schindler et al. (2006) for the Western blot analysis of xCT, EAAC1, LAT1, GLAST, GLT-1, NR2A, and NR2B. Frozen tissues were homogenized in 20 volumes of CLB buffer containing 10 mM HEPES, 10 mM NaCl, 1 mM KH_2_PO_4_, 5 mM NaHCO_3_, 5 mM EDTA, 1 mM CaCl_2_, 0.5 mM MgCl_2_, 1 mM PMSF, and inhibitor protease cocktail. The homogenates were centrifuged at 6,300× *g* for 15 min at 4°C. The supernatants were recovered and centrifuged at 100,000× *g* for 30 min at 4°C. The pellets were finally suspended in 150 μl of 40 mM Tris–HCl at pH 9.5, 8 M urea, and 4% (*w*/*v*) Triton X-100. Protein concentrations were quantified using a Pierce BCA protein assay kit (Thermo Scientific, Rockford, IL, USA).

The samples (20–40 μg of protein per well) were subjected to sodium dodecyl sulfate polyacrylamide gel electrophoresis (SDS-PAGE) and transferred onto nitrocellulose membranes (Bio-Rad Laboratories, Germany). The membranes were blocked with Tris-buffered saline (TBS) containing 5% Blotto and 0.1% Tween-20 and incubated overnight at 4°C with the appropriate primary antibodies (CBS, 1:1,000; xCT, 1:2,000; EAAC1, 1:2,000; LAT1, 1:1,000; GLAST, 1:2,000; GLT-1, 1:2,000). The blots were probed with mouse anti-β-tubulin (1:5,000) after stripping, which was used as a loading control. The protein bands were visualized with appropriate horseradish peroxidase (HRP)-linked secondary antibodies using the ECL Prime Western Blotting Detection Reagent (GE Healthcare Bio-Sciences, Pittsburgh, PA, USA). Images were captured, and densitometric analysis was performed with ImageJ software version 1.46r software (US National Institutes of Health, Bethesda, MD, USA).

### Quantitative RT-PCR Analysis of *nfe2l2* and *ikkbα*

Total RNA from the cortex of mice was isolated using TRIzol (Invitrogen, Carlsbad, CA, USA). The RNA integrity of the samples was assessed by electrophoresis in 1% agarose gels. The absorbance indices *A*_260/280_ and *A*_260/230_ were used to assess the purity of the isolated RNA. RNA concentrations were determined by measuring the absorbance at 260 nm. One microgram of total RNA from successful individual samples was reverse transcribed to complementary DNA (cDNA) at 37°C using Moloney murine leukemia virus (M-MLV) reverse transcriptase and oligo(dT)15 primer (Promega, Madison, WI, USA). For quantitative PCR analysis, the cDNA of individual samples was diluted to 20 ng of input total RNA in a reaction mixture containing 0.5 μM of each respective forward and reverse primer and 1× KAPA SYBR FAST Universal Mix (Kapa Biosystems, Cape Town, South Africa). A Rotor-Gene Q PCR cycler (Qiagen GmbH, Hilden, Germany) was used to quantify PCR products. The PCR conditions were an initial heating at 94°C for 3 min, followed by 40 cycles of 94°C for 1 s, 63°C for 10 s, and 72°C for 12 s. Finally, melting curves were generated from 73 to 93°C for each PCR run. Succinate dehydrogenase (*SDHA*) was used as the reference gene. The mean amplification efficiency calculated in each PCR run for *nfe2l2*, *ikkbα*, and *SDHA* from fivefold dilution curves was 1.017 ± 0.035 (standard deviation), with *R*^2^ being 0.993 ± 0.005 (all above 0.982). The primers used were as follows: 5′-caccagtggatccgccagcta-3′ and 5′-tatccagggcaagcgactca-3′ for *nfe2l2* (Valdovinos-Flores et al., 2019); 5′-aaatctccagatgctacccgagag-3′ and 5′-ataatgtcagacgctggcctccaa-3′ for *iκκbα* (Valdovinos-Flores and Gonsebatt, 2013); and 5′-caaatgctggagaagaatcggt-3′ and 5′-catcgacttctgcatgtttaggc-3′ for *SDHA*. The results were analyzed using the 2^−ΔΔCT^ method (Livak and Schmittgen, 2001) and expressed as the mean normalized *nrf2* and *ikkbα* values ± SD.

### Measurement of Hydrogen Sulfide Production

We followed the protocol described by Hine and Mitchell (2017) with some adaptations. Briefly, ~100 mg of frozen cortex or liver samples was homogenized in 250 μl of ice-cold lysis buffer (25 mM Tris Base, 1 mM DTT, 5% glycerol, 1% Triton) after homogenization. The samples were placed at 37°C for 5–10 min and frozen again in dry ice for 2–3 min. The freeze–thaw cycle was repeated three times. Supernatants (~200 μl) were removed after the homogenates were centrifuged at 5,000× *g* for 5 min at 4°C. Protein concentration was determined using the Pierce BCA Protein Assay kit (Thermo Fisher Scientific, Rockford, IL, USA). Filter papers were soaked in 20 mM lead (II) acetate trihydrate for 20 min and subsequently dried in an oven set at 110°C for 30 min. The assay was run in a 96-well plate with 150 μl of the L-cysteine [100 mM in phosphate-buffered solution (PBS)] and pyridoxal 5′-phosphate (PLP; 10 mM in PBS) working solutions and 0–500 μg of sample protein. Liver samples from untreated animals were used as positive controls. The dry lead acetate-embedded filter paper was placed directly over the 96-well plate and incubated at 37°C for 6 h for liver homogenates and 16 h (overnight) for cortex homogenates. The images were scanned, and densitometric analysis was performed with ImageJ software version 1.46r software (US National Institutes of Health, Bethesda, MD, USA).

### Data Analysis

The data are expressed as the mean ± standard error. The number of animals tested is indicated in each case. One- and two-way analysis of variance (ANOVA) were used to assess statistical significance followed by Tukey’s *post hoc* test, as indicated in the corresponding figures. A *P* < 0.05 was considered statistically significant in all cases.

## Results

### GSH Levels in the Cortex, Striatum, and Cerebellum Are Affected by iAs

**Figure 1:**
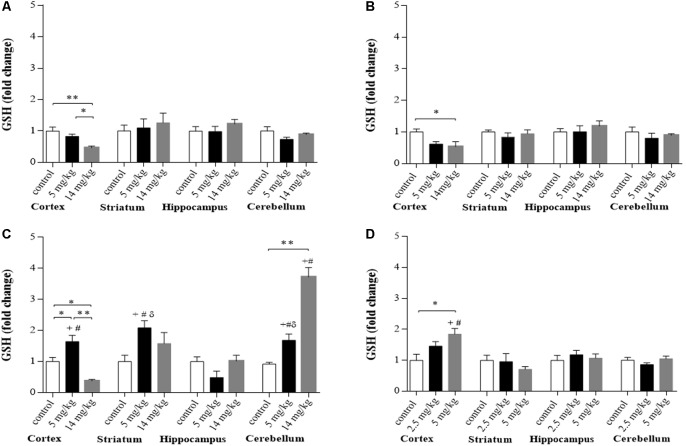
Glutathione (GSH) levels in different brain regions of mice treated i.p. with 0 (controls), 2.5, 5, or 14 mg/kg NaAsO_2_. Normalized levels of GSH ratios in animals treated with 5 and 14 mg/kg at 2 h **(A)**, 6 h **(B)**, and 24 h **(C)** and animals treated with 0, 2.5, or 5 mg/kg for 9 days **(D)**. Data in graphs represent the mean ± SE, *n* = 6. The data were analyzed using one and two-way analysis of variance (ANOVA) with Tukey’s *post hoc* analysis. Different superscript symbols above each column indicate statistically significant differences; *P ≤ 0.05 and **P ≤ 0.01 for dose-related differences; plus symbol denotes differences from the 2 h group, number symbol denotes differences from the 6 h and delta symbol denotes differences from 9-day treatment. Significance was accepted at *P* ≤ 0.05. Significance was accepted at *P* ≤ 0.05.

Mice with prolonged exposure to iAs show increased levels of GSH (Ramos-Chávez et al., 2015). Here, significantly decreased levels of GSH were observed in the brain cortex region at 2 and 6 h for both doses of iAs and at 24 h in the animals treated with the highest dose (Figures 1A–C), while animals treated with 5 mg/kg showed upregulated GSH synthesis at 24 h (Figure 1C) in both the cortex, striatum, and cerebellum, suggesting that GSH was actively synthesized in these regions at that time. Animals treated with the highest dose also showed increased levels of GSH in the cerebellum at 24 h (Figure 1C). We measured the levels of GSH in the cortex of animals that received iAs for 9 days and observed a similar dose-related increase in the GSH levels in the cortex (Figure 1D). In the cerebellum, the increase in GSH levels at 9 days was higher than that observed at 2 and 6 h, respectively.

### Expression of xCT and EAAC1 in the Cortex Is Related to GSH Synthesis and to GLT-1 and GLAST Upregulation

**Figure 2:**
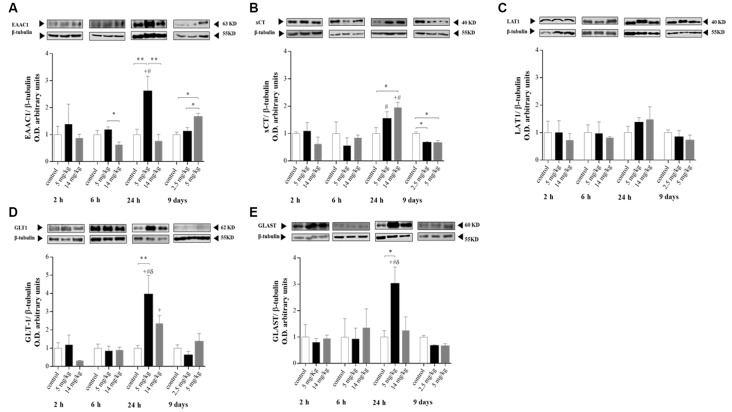
Protein levels of the amino acid transporters excitatory amino acid transporter 3 (EAAT3; **A**), xCT **(B)**, L-type amino acid transporter 1 (LAT1; **C**), glial glutamate transporter 1 (GLT-1; **D**), and glutamate/aspartate transporter (GLAST; **E**) in the cortex of mice treated with 0, 2.5, 5, or 14 mg/kg NaAsO_2_. Changes in the protein levels in the enriched membrane fraction were evaluated by Western blot analysis at 2, 6, 24 h and 9 days after the last NaAsO_2_ administration. Bars represent the densitometric analysis of the protein bands normalized to β-tubulin, mean ± SE, *n* = 6. The data were analyzed using one and two-way ANOVA with Tukey’s *post hoc* analysis. Different superscript symbols above each column indicate statistically significant differences; *P ≤ 0.05, **P ≤ 0.01 for dose-related differences; plus symbol denotes differences from the 2 h group, number symbol denotes differences from the 6 h group, and delta symbol denotes differences from 9 days group. Significance was accepted at *P* ≤ 0.05.

At the times tested, the cortex was the brain region where significant changes in protein expression were observed. No changes in the expression of LAT1, EAAC1, or xCT were observed at 2 and 6 h after treatment (Figures 2A–C). However, at 24 h, enhanced expression of EAAC1 and xCT was observed in the 5 and 14 mg/kg iAs-treated groups, respectively (Figures 2A,B). The upregulation of EAAC1 expression was maintained in the 9-day 5 mg/kg (Figure 2A) semichronic iAs treatment group, while xCT expression in both chronic treatment groups decreased (Figure 2B).

The modulation of EAAC1 expression in both the 5 mg/kg group 24 h after injection and in the 9-day 5 mg/kg chronic iAs treatment group was directly related to the modulation of GSH levels (Figures 1, 2A), suggesting the importance of cystine uptake by EAAC1 into the cells to synthesize GSH, mainly in neurons.

No changes in the expression of GLT-1 and GLAST were observed at 2 or 6 h or in the 9-day treatment group (Figures 2D,E). However, enhanced expression of GLT-1 and GLAST (Figures 2D,E) was associated with xCT and EAAC1 upregulation at 24 h in 5 mg/kg iAs-treated animals (Figures 2A,B).

### Modulation of NR2A and NR2B Expression

**Figure 3:**
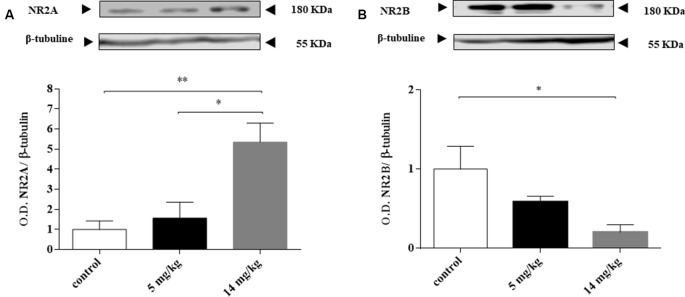
Inorganic arsenic (iAs) effect on the protein levels of the *N*-methyl-D-aspartate receptor (NMDAR) subunits NR2A **(A)** and NR2B **(B)** in the cortex of mice treated with 0, 5, or 14 mg/kg NaAsO_2_. Changes in the protein levels in the enriched membrane fraction were evaluated by Western blot analysis 24 h after the last NaAsO_2_ administration. Bars show the densitometric analysis of the protein bands normalized to β-tubulin, mean ± SE *n* = 6. The data were analyzed using one-way ANOVA with Tukey’s *post hoc* analysis. Different superscript symbols above each column indicate statistically significant differences; *P ≤ 0.05, **P ≤ 0.01.

Chronic exposure to iAs was associated with increased extracellular glutamate and the negative modulation of the expression and activity of the NMDAR subunits NR2A and NR2B (Luo et al., 2009, 2012; Ramos-Chávez et al., 2015; Nelson-Mora et al., 2018). In the cortex, NMDAR acts as a detector for activity-dependent plasticity and associative learning (Hasan et al., 2013). We decided to evaluate the expression of these subunits at 24 h because the expression of GLT-1 and GLAST transporters was enhanced at this timepoint. NR2A subunit expression was increased, while NR2B subunit levels were decreased (Figure 3) only in those animals receiving the highest dose (14 mg/kg group).

### CBS Protein Expression

**Figure 4:**
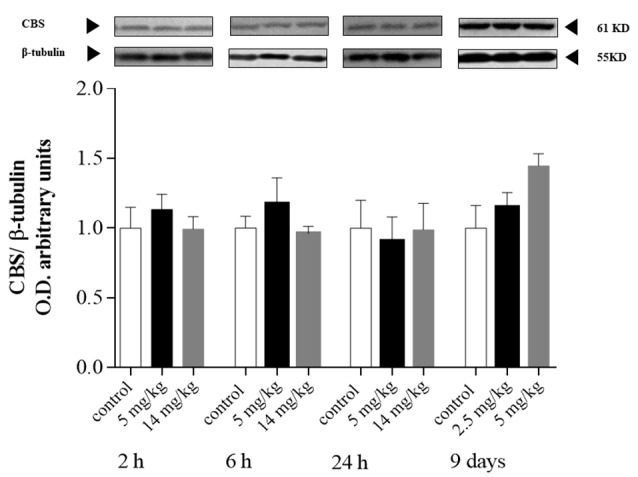
Protein levels of cystathionine-β-synthase (CBS) in the cortex of mice treated with 0, 2.5, 5, or 14 mg/kg NaAsO_2_. Changes in the protein levels of total lysate homogenates were evaluated by Western blot analysis at 2, 6 h or 24, 24 h after the last NaAsO_2_ administration. Bars show the densitometric analysis of the protein bands normalized to β-tubulin, mean ± SE, *n* = 6. The data were analyzed using one-way ANOVA with Tukey’s *post hoc* analysis.

CBS is the dominant enzyme of the transsulfuration pathway in astrocytes, which provides cysteine for GSH synthesis (Lee et al., 2009; McBean, 2012; Niu et al., 2015). In addition, Coppin et al. (2008) showed that iAs induces GSH synthesis and the transcription of CBS in cultured human cells. In our model, CBS protein levels (Figure 4) were not upregulated in the cortex at any of the times explored, even though GSH levels increased at 24 h in this region (Figure 1).

### Increased Transcription of *nfe2l2* and *iκκbα* at 6 h Suggests Nrf2 and NFκB Activation Is Associated with Diminished GSH Pools and Upregulated Amino Acid Transporters

**Figure 5:**
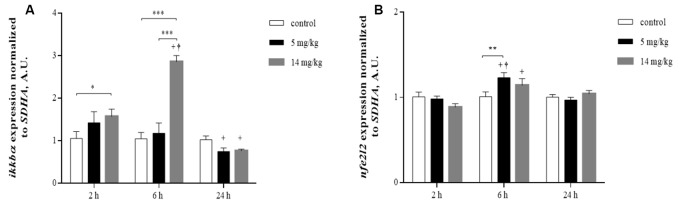
Inorganic arsenic (iAs) effects on the messenger RNA (mRNA) levels of *ikkbα* **(A)** and *nfe2l2* **(B)** in the cortex of mice treated with 0, 5, or 14 mg/kg NaAsO_2_. For real-time PCR, total RNA was extracted from the brain cortex as described in “Materials and Methods” section. The data were normalized to the *SDHA* mRNA expression. The vertical bars indicate the mRNA levels relative to the control group. The bars represent the mean ± SE (*n* = 7). Different superscript symbols above each column indicate statistically significant differences; *P ≤ 0.05, **P ≤ 0.01, ***P < 0.001. Plus symbol denotes differences from the 2 h group, dagger symbol denotes differences from the 24 h group, two-way ANOVA with Tukey’s *post hoc* analysis. Significance was accepted at *P* ≤ 0.05.

Putative nuclear factor erythroid 2-related factor 2 (Nrf2) and nuclear factor kappa B (NFκB) binding sites have been reported for xCT, LAT, GLT-1, and GLAST (Valdovinos-Flores and Gonsebatt, 2012; Martinez-Lozada et al., 2016). In addition, there is evidence that NFκB inhibitors diminish the expression of EAAC1 in rats (Tai et al., 2008). Nrf2 and NFκB upregulate the transcription of the *nfe2l2* and *iκκbα* genes, respectively (Valdovinos-Flores and Gonsebatt, 2013; Tonelli et al., 2018) and are considered redox-sensitive switches that activate cellular responses to oxidative stress (Moldogazieva et al., 2018). To investigate whether the activation of these transcription factors was associated with increased amino acid transporter expression and increased GSH levels at 24 h, we measured the transcription of the *nfe2l2* and *iκκbα* genes by quantitative reverse transcription PCR (RT-PCR) at 2, 6 and 24 h in the cortex region. A significant increase in the levels of *iκκbα* messenger RNA (mRNA) was observed at 6 h, while only *nfe2l2* mRNA was significantly increased by the 5 mg/kg dose at this time. At 24 h, the transcription of both genes returned to control levels or below control levels (Figures 5A,B), suggesting the participation of both transcription factors in this acute response to iAs.

### H_2_S Production

**Figure 6:**
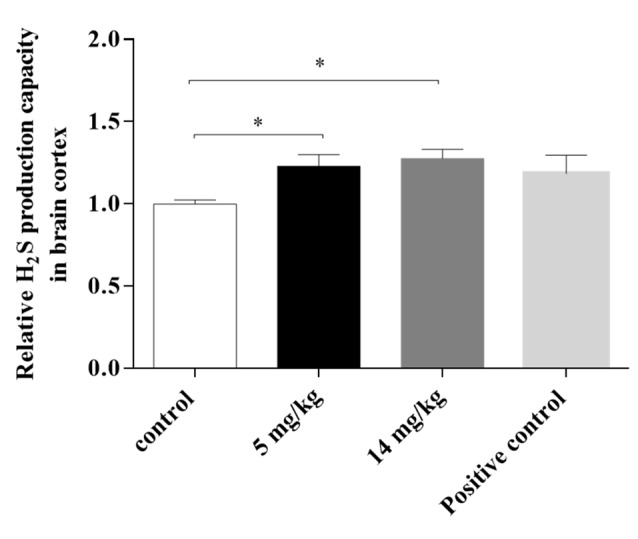
H_2_S production capacity in the brain cortex of mice treated with 5 or 14 mg/kg NaAsO_2_ at 24 h. Changes in H_2_S production capacity were evaluated by a lead sulfide method. Bars show the IntDen function measurement normalized to the respective control group, mean ± SE, *n* = 7. Untreated mouse liver tissue was used as a positive control. The data were analyzed using one-way ANOVA with Tukey’s *post hoc* analysis. Different superscript symbols above each column indicate statistically significant differences; *P ≤ 0.05.

Cortex homogenates were assayed for H_2_S production at 24 h, since CBS was not upregulated at this time. H_2_S production has been directly related to the activity of CBS in the brain, providing L-cys for GSH synthesis *via* transsulfuration from methionine sources (Kandil et al., 2010). IAs-treated animals (5 and 14 mg/kg) showed a significantly higher production of the neuromodulator than did the control animals (Figure 6), suggesting that enhanced CBS (and or CSE) enzymatic activity is associated with the increased GSH synthesis observed in the 5-mg/kg treated animals.

## Discussion

In this acute exposure study, we observed that iAs exposure altered GSH levels and the disposition of key amino acids and their transporters in the cortex and cerebellum. At 24 h, a redox response, probably activated by Nrf2 and NFκB (Figure 5), was observed mainly in the cortex region with increased GSH levels that were associated with the increased production of H_2_S and the upregulation of xCT, GLT-1, GLAST, and EAAC1. In addition, the expression of NMDA subunits was altered at the highest dose employed.

Sato et al. (2001) reported a putative NFκB binding site in the 5′-flanking region of the xCT gene. In addition, NFκB binding sites have been reported for GLT-1 and GLAST (Martinez-Lozada et al., 2016; Pajarillo et al., 2019), and NFκB inhibitors diminished EAAC1 expression (Tai et al., 2008). Evidence shows that both transcription factors are key molecules in redox signal transduction pathways (Valdovinos-Flores and Gonsebatt, 2012; Moldogazieva et al., 2018).

The effects of iAs could be observed as early as 2 and 6 h when GSH levels were diminished significantly, mainly in the cortex region (Figure 1A), which could activate the redox response observed at 6 h by the activation of Nrf2 and NFκB (Figure 5). Valdovinos-Flores and Gonsebatt (2013) observed significant transcription of *ngfb* at 2 h after a 14-mg/kg iAs i.p. injection, which was associated with the activation of the TrkA/Akt/NFκB signaling pathway in the liver but not in the striatum of mice (Valdovinos-Flores et al., 2019). However, systemic activation of this pathway could induce GSH synthesis in other brain regions such as the cortex and cerebellum at 24 h and 9 days (Figures 1C,D). Increased levels of GSH were observed in mouse brain homogenates (Limón-Pacheco et al., 2007) and in the cerebellum at 2 h after the administration of L-buthionine-*S*-*R*-sulfoximine (BSO), a systemic inhibitor of GSH synthesis, which diminished GSH levels in the liver and kidneys (Limón-Pacheco et al., 2007; Valdovinos-Flores and Gonsebatt, 2013; Garza-Lombó et al., 2018b).

CD1 mice that received iAs for 9 days showed GSH reductase inhibition in the liver and brain at 10 mg/kg (Rodríguez et al., 2005). This enzyme is key for the reduction in oxidized GSH. Thus, we used lower doses to compare the early effects on GSH and amino acid transporters with those observed after a prolonged exposure. Similar to what has been observed in mice exposed during gestation, the augmentation of GSH levels in the brain was directly related to the expression of amino acid transporters involved in the import of cystine/cysteine for GSH synthesis, such as xCT in astrocytes and EAAC1 in neurons (Figures 1A–D, 2A,B; Ramos-Chávez et al., 2015; Nelson-Mora et al., 2018). However, the upregulation of EACC1 was maintained only in the group of mice that received the higher dose of iAs for 9 days (5 mg/kg), which was positively associated with increased levels of GSH in that region (Figures 1D, 2A). These observations suggest that the participation of EACC1 might be crucial for maintaining GSH homeostasis after iAs exposure. In this respect, De Bundel et al. (2011) demonstrated that xCT knockout mice do not have a lower hippocampal GSH content or increased oxidative stress. In contrast, mice lacking EAAC1 (Aoyama et al., 2006) have decreased neuronal GSH contents, accompanied by increased neuronal oxidative stress markers and severe spatial reference memory deficits with aging. The L-cys for GSH synthesis in this case, could also be provided by the transsulfuration pathway, although we did not test for the production of H_2_S at this time point.

GLT-1 and GLAST expression was only increased in the cortex at 24 h, at the same time as xCT upregulation. The transporters GLT-1 and GLAST are considered the primary astrocyte regulators that mediate extracellular glutamate clearance in the CNS (Amara and Fontana, 2002). The promoter regions of both GLT-1 and GLAST contain multiple NFκB binding sites (Pajarillo et al., 2019), while for xCT, Nrf2 and NFκB binding sites have been proposed (Valdovinos-Flores and Gonsebatt, 2013). Thus, both the export (xCT) and uptake (GLT-1 and GLAST) of glutamate could be modulated by these transcription factors, which would diminish extracellular glutamate release by the upregulation of xCT at this same time (Figures 2D,E). Previously, we observed that the gestational and chronic exposure of mice to iAs resulted in increased glutamate levels in the hippocampus associated with the upregulation of xCT and the downregulation of GLT1 and GLAST (Ramos-Chávez et al., 2015; Nelson-Mora et al., 2018).

With respect to altered glutamate receptor subunit expression (Figure 3), mechanistic studies in rodent models chronically exposed to iAs (Huo et al., 2014; Ramos-Chávez et al., 2015) showed that the negative modulation of the NMDAR subunits NR2A and NR2B was associated with the upregulation of xCT. In our case, the altered expression of NMDA subunits (Figure 3) agrees with the upregulation of xCT at 24 h, suggesting that the increased extracellular glutamate exported by the increased xCT could downregulate the NR2B subunit, similar to what was observed in the hippocampus of mice exposed during gestation (Ramos-Chávez et al., 2015). The AMPA receptor subunits GLUA1 and 2 were also tested, but we did not observe changes (data not shown). A similar observation was made by Nelson-Mora et al. (2018) when AMPA modulation was reported at the hippocampus but not in the cortex of mice gestationally exposed to iAs.

The cysteine required for GSH synthesis and iAs metabolism (Garza-Lombó et al., 2019) can be provided through the transsulfuration pathway, which links the SAM pathway and GSH production, both main factors involved in iAs methylation. Coppin et al. (2008) showed that the adaptation of RWPE-1 cells to arsenic includes increased mRNA expression of CBS and GSH production genes, which results in a fivefold increase in GSH. In addition, the presence of CBS polymorphisms in human populations might influence arsenic metabolism (Porter et al., 2010). Here, we did not observe changes in the expression of CBS protein (Figure 4) and mRNA in the cortex at 24 h (data not shown), but we observed increased H_2_S production (Figure 6) in cortex and liver homogenates (see Supplementary Figures S1, S2), suggesting that the activity of the transsulfuration pathway was increased. Since both CBS and CSE are modulated by NFκB (Huang and Moore, 2015), it is possible that the increased production of H_2_S could also be due to CSE.

In addition, H_2_S is a gasotransmitter that can pass through cell membranes, modulating cellular targets at physiological levels by S-sulfhydration (Wang et al., 2014). The increased generation of H_2_S at 24 h might also participate in the modulation of GLT-1 expression observed at this time, increasing the uptake of glutamate interchanged by the input of cystine during xCT overexpression (Lu et al., 2008; Nelson-Mora et al., 2018). It has been reported that H_2_S activates K_ATP_ channels by the S-sulfhydration of the Cys6 and Cys26 residues of the rvSUR1 subunit of the K_ATP_ channel complex (Jiang et al., 2010). The upregulation of GLT-1 could also be due to the activation of K_ATP_ channels by H_2_S since Sun et al. (2008) reported that K_ATP_ activators in astrocytes can upregulate glutamate transporters. It has been suggested that, at physiological levels, H_2_S can exert neuroprotective effects, whereas high concentrations of H_2_S may cause neurotoxicity in part by enhancing NMDAR-mediated calcium overload. In our case, we observed an upregulation of NR2A and downregulation of NR2B subunit expression but did not measure NMDA activity, although mice chronically exposed to iAs showed impaired long-term potentiation (LTP) induction in the hippocampus (Nelson-Mora et al., 2018).

Exposure to hazardous environmental metalloids such as iAs throughout our lifetime is almost unavoidable because not only drinking water but also many types of foods might be contaminated with different levels of iAs. Few studies have explored the acute effects of iAs exposure, which are important to elucidate since the early effects were not homogeneous among different brain regions and because iAs exposure compromised antioxidant levels and important amino acid disposition such as glutamate, cysteine, and methionine. The impact of these changes in the CNS might contribute to premature aging and/or to earlier neurodegenerative manifestations. Our results can help in the development of adequate preventive strategies, such as appropriate diets and the development of effective agonists.

## Data Availability Statement

All datasets generated for this study are included in the article/Supplementary Material.

## Ethics Statement

The animal study was reviewed and approved by Institute of Biomedical Research CICUAL committee.

## Author Contributions

DS-A designed the study, performed the experiments, and prepared the manuscript draft. PP helped with the RT-PCR study and LR-C helped with the Western blot analysis. WG-A and AP-A performed the hydrogen sulfur determination and contributed in the discussion. MG designed the study, applied for approval from the Research Ethics Board, and reviewed the manuscript draft.

## Conflict of Interest

The authors declare that the research was conducted in the absence of any commercial or financial relationships that could be construed as a potential conflict of interest.
