# Supplementary figures and images for: Early Neurotoxic Effects of Inorganic Arsenic Modulate Cortical GSH Levels Associated With the Activation of the Nrf2 and NFκB Pathways, Expression of Amino Acid Transporters and NMDA Receptors and the Production of Hydrogen Sulfide
Source: Front Cell Neurosci. 2020 Feb 25;14:17. doi: 10.3389/fncel.2020.00017 (PMC7065714; doi:10.3389/fncel.2020.00017)

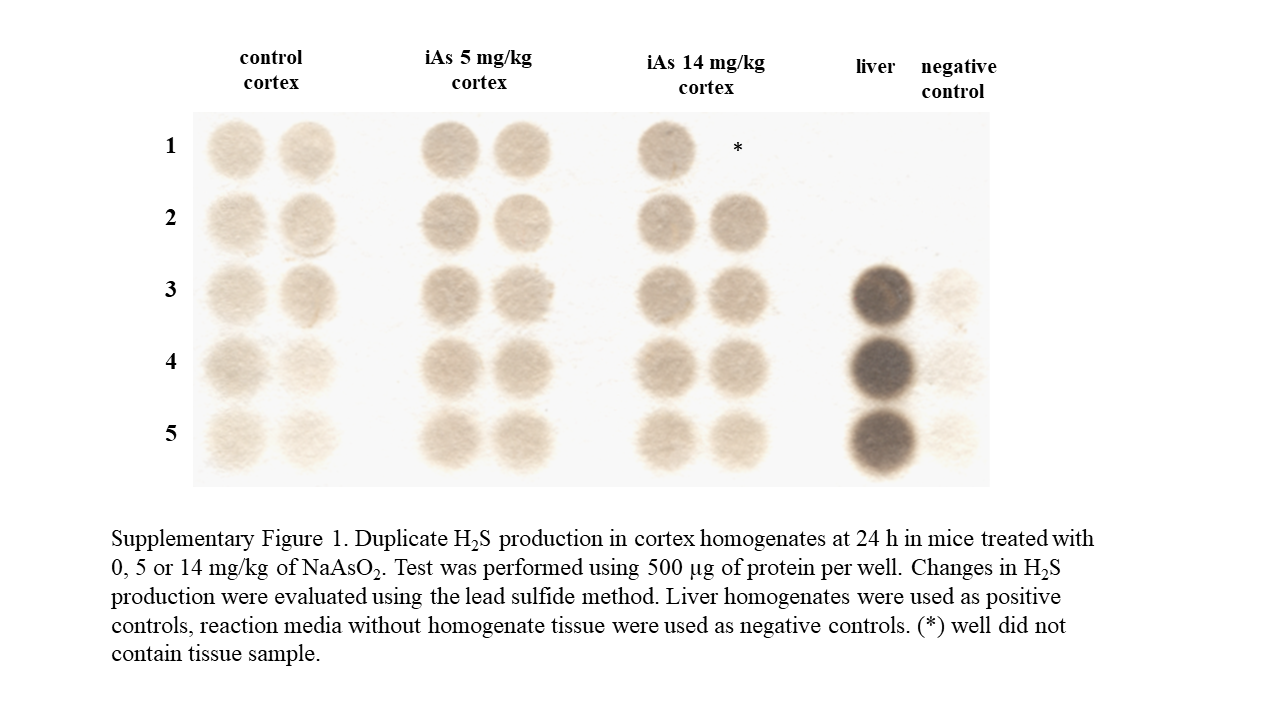

Supplement: Supplementary file 1 [file Image_1.TIF]

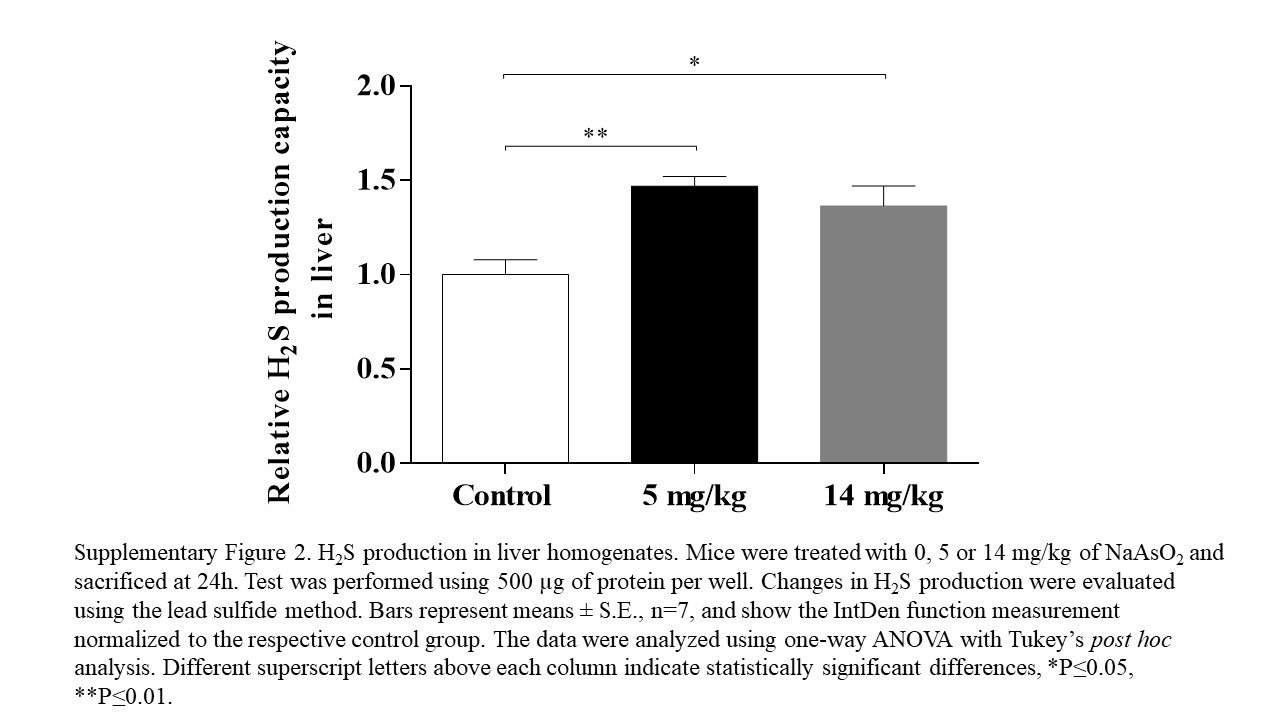

Supplement: Supplementary file 2 [file Image_2.tif]
